# Investigating the Adsorption and Corrosion Protection Efficacy and Mechanism of *Marjoram* Extract on Mild Steel in HCl Medium

**DOI:** 10.3390/molecules30020272

**Published:** 2025-01-11

**Authors:** Malika Sabiha, Younes Kerroum, Maha El Hawary, Maria Boudalia, Abdelkbir Bellaouchou, Othmane Hammani, Hatem M. A. Amin

**Affiliations:** 1 Laboratory of Materials, Nanotechnology and Environment, Faculty of Sciences, Mohammed V University, Rabat 1014, Morocco; sabihamalika@gmail.com (M.S.); maria.boudalia@yahoo.fr (M.B.);; 2 Ingeniería Electroquímica y Corrosión (IEC), Instituto Universitario de Seguridad Industrial, Radiofísica y Medioambiental (ISIRYM), Universitat Politècnica de València, 46022 Valencia, Spain; 3 Faculty of Sciences, Moulay Ismail University, Meknes 52202, Morocco; othmane.hammani@hotmail.com; 4 National Center for Scientific and Technical Research (CNRST), Rabat 10102, Morocco; 5 Chemistry Department, Faculty of Science, Cairo University, Giza 12613, Egypt; 6 Faculty of Chemistry, University of Duisburg-Essen, 45141 Essen, Germany

**Keywords:** green inhibitors, low-carbon steel, adsorption, plant extract, essential oil, corrosion inhibition, EIS

## Abstract

In recent years, the anti-corrosive properties of natural extracts as environmentally friendly inhibitors have gained considerable interest. This study evaluates the potential of *Marjoram* (*Origanum majorana* L.) essential oil (*OML*), collected from Salé, Morocco, as a corrosion inhibitor for mild steel in 1 M HCl medium. The protection performance of *OML* was assessed using various electrochemical techniques, including potentiodynamic polarization (PDP) and electrochemical impedance spectroscopy (EIS), as well as the weight loss method. The influence of *OML* concentration and temperature on the inhibition performance were investigated. *OML* demonstrated pronounced inhibitory benefits via increasing the corrosion resistance of mild steel in the corrosive HCl solution, thus reducing the corrosion rate to 0.11 mg cm^−2^ h^−1^ and increasing the inhibition efficiency to 87.1% at an inhibitor concentration of 500 ppm. PDP confirmed that the inhibitor works as a mixed-type inhibitor with cathodic supremacy. EIS revealed that the charge transfer mechanism is the main controlling factor for the corrosion process. The thermodynamic parameters suggested a key role of *OML* physisorption in inhibition, following the Langmuir isotherm. Importantly, SEM and EDX analyses suggested the formation of a protective layer of the extract onto the steel surface, which shields the surface from corrosive species. This is owed to the functional group-rich phytochemicals of *OML*. Therefore, the development of bio-based corrosion inhibitors is not only a step towards more eco-friendly industrial practices, but also meets the growing demand for sustainable materials in a world with constrained resources.

## 1. Introduction

Metals and their alloys have been increasingly used in many technologies and structures. However, they are prone to corrode due to their interaction with their surroundings [1]. The process of corrosion is an inevitable and natural phenomenon that causes damage to materials, especially metals such as steel, through reactions with their environment. This damage poses major challenges for steel-dependent sectors such as the construction, transportation, and energy industries, and leads to economic losses, safety risks, and environmental problems [2]. Therefore, it is necessary to implement corrosion protection methods and consequently ensure the durability of metallic infrastructures, enhance safety, and reduce environmental and economic impacts. The use of corrosion inhibitors has attracted great attention due to the simplicity, adaptability, and cost-effectiveness of this approach, and therefore inhibitors are widely used in the machinery, refining, oil and gas extraction, chemical, and energy industries [3,4,5]. Though they have high corrosion prevention efficacy in a range of corrosive media, many of the used synthetic organic and inorganic corrosion inhibitors are toxic and hazardous to the environment [6]. From the point of view of environmental compatibility and human health protection, there has been a growing interest in sustainable and green corrosion inhibitors.

In this context, natural extracts such as plant extracts are increasingly being explored as eco-friendly and sustainable corrosion inhibitors, offering a green and cost-effective option to corrosion inhibition [7,8]. Plant extracts contain a wealth of bioactive compounds, such as alkaloids, flavonoids, tannins, and terpenoids, which interact in different ways with metal surfaces to prevent corrosion [9,10]. We note that the bioactive compounds present in plant extracts can be equally effective as synthetic inhibitors. Recent studies [11,12,13] have highlighted the potential of medicinal plants as green inhibitors, especially when metals are exposed to aggressive environments, such as acidic solutions. In particular, natural substances from plant extracts and essential oils were investigated and showed promising protection performance [14,15,16]. For example, an extract of *Chamaerops humilis* fruit waste demonstrated a high corrosion protection efficacy for low-carbon steel in HCl medium [16]; this natural inhibitor functions as a mixed-type inhibitor with a maximum inhibition efficiency of 93% at a concentration of 500 ppm. In our previous work, using a methanolic extract of parsley with carbon steel C37 in 1 M HCl, a concentration-dependent inhibition efficiency was revealed with an inhibition efficiency peaking at 92% at 1000 ppm [15].

Particularly in acidic environments, metals and alloys have high susceptibility to corrosion. Most mechanisms of inhibition involve the adsorption of a variety of phytochemicals on the substrate surface, forming a protective film. This film functions as a physical barrier that shields the metal surface from the attack of corrosive species, such as chloride, hydroxide, moisture, oxygen, and acidic environments [6,17]. This adsorption-driven protection not only prevents direct interaction (either chemical and/or physical) between the steel and corrosive species, but also can alter the electrochemical reactions that lead to corrosion. Previous studies have shown that plant essential oils exhibit potential inhibition efficiency in different media, and these inhibitors can suppress anodic, cathodic, or both reactions in the corrosion process [18,19,20,21].

To maximize the efficiency and sustainability of plant extract inhibitors, improving the extraction process is an important measure. For this, the influence of the extraction method on corrosion-inhibiting properties has been studied For example, six different extraction solvents with increasing polarity were tested for the same sample of *Sargassum*; the results showed that cold maceration from chloroform extract exhibits the best inhibitory efficiency of 92.0% [22]. In addition, the effectiveness of natural substances as corrosion inhibitors can be attributed to their chemical and structural properties, which are often characterized by the presence of compounds containing π bonds and heteroatoms, which facilitate their interaction with the metal surface, resulting in the formation of a protective layer that reduces the corrosion rate [14]. Recently, the anticorrosive efficiency of the extracts of five different herbal medicines (namely, Yarrow, Wormwood, *Maurorum*, *Marjoram*, and *Ribes rubrum*) was evaluated for mild steel in 1 M HCl solution [23]. The results proved that the protonated organic compounds in the extracts have high adsorption capacity on the electrode surface.

One such interesting plant, *Origanum majorana* L., also known as sweet marjoram or *Marjoram*, is a member of the Origanum species of the *Lamiaceae* family and is well known for its various properties in the pharmaceutical field. Native to the Mediterranean region, its essential oil has gained scientific attention for its diverse properties. Beyond the therapeutic use of *Marjoram* extract, recent research has highlighted its potential as a bio-based corrosion inhibitor. For instance, *Origanum* essential oils were used as an ecofriendly corrosion inhibitor for mild steel in HCl solution; however, a different *Origanum* species, *Origanum vulgare*, was used and a much higher extract concentration of 2000 ppm was used to achieve an efficiency of about 87% [24]. Additionally, the electrochemical investigations were lacking.

*Marjoram* extracts have demonstrated potential corrosion inhibition, providing a sustainable option for metal protection in various environments [25,26,27]. In our recent work, we demonstrated the promising bioactivity and enhanced anticorrosive efficacy of an essential oil extract of *Origanum majorana* L. from the region Errachidia, Morocco [28]. However, it is well established that the constituents and properties of plant extracts and oils can be strongly influenced by climate, soil composition, altitude, cultivation practices, and genetic variations [29,30,31]. Such changes in composition or concentration are expected to alter the nature of their interaction with metal surfaces. This warrants further investigation of the factors influencing the extract’s chemical profile and anticorrosive properties, such as the influence of the region of origin of *EO-OML* extracts prepared using the same extraction method.

In this work, an essential oil of *Origanum majorana* L. (*OML*) from the Salé region, Morocco, was prepared to investigate its corrosion protection performance for mild steel in HCl solution. Its anticorrosive properties were evaluated using various methods, including weight loss, potentiodynamic polarization, and electrochemical impedance spectroscopy (EIS). The effect of independent parameters such as inhibitor concentration and temperature on corrosion resistance was studied. The results were compared with the published data on *OML* extract from the Errachidia region. Additionally, the thermodynamic parameters of corrosion were determined. The surface morphology and composition of the tested steel sample were examined in the presence and absence of the *OML* inhibitor using scanning electron microscopy coupled with energy-dispersive X-ray analysis (SEM-EDX). This helped us to understand the inhibition mechanism.

## 2. Results and Discussion

### 2.1. Weight Loss (WL) Assay

To quantify the anticorrosion activity of *OML* oil on mild steel in HCl solution, the weight loss method was initially utilized. The tests were performed by immersing the mild steel samples in 100 mL of 1 M HCl solution for 6 h in the absence and presence of *OML*, with different concentrations of 200, 300, 400, and 500 ppm at 303 K. The weight loss in the specimen after immersion was measured, from which the *CR* (mg cm^−2^ h^−1^) and corresponding inhibition efficiency were calculated, as presented in Table 1.

Table 1 shows that the inhibition efficiency increases as the inhibitor concentration increases from 200 ppm to 500 ppm, indicating the dependance of the corrosion inhibition efficacy on the amount of *OML* extract in the solution. The *OML* inhibitor showed a maximum inhibition efficiency of 87% at an optimum concentration of 500 ppm. This tendency could indicate that higher *OML* content can protect the surface of mild steel more effectively and thus protect the surface from attack by corrosive substances.

In general, when mild steel is exposed to acidic environments, it is susceptible to the corrosion process, which involves electrochemical reactions leading to material loss; active sites are created by the oxidation of iron, forming iron ions. The presence of an inhibitor attenuates this process by various mechanisms, such as modifying the electrochemical reactions occurring on the metal surface and creating a protective layer that reduces the availability of ion exchange, in particular by decreasing the contact of the metal with corrosive agents such as proton ions and chloride ions. The high anticorrosive efficiency of our extract supports the study of Sobhi [25] on an aqueous extract of *OML*, which showed promising results for inhibiting the corrosion of zinc in 1 M HCl.

### 2.2. Potentiodynamic Polarization Measurement (PDP)

**Figure 1:**
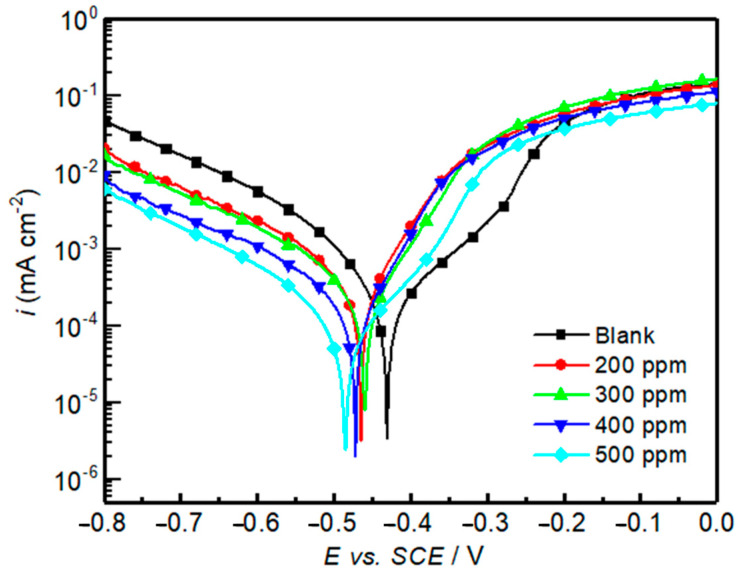
Potentiodynamic polarization curves of mild steel in 1 M HCl using different *OML* concentrations at 303 K.

To further analyze the potential of *OML* in inhibiting the corrosion of mild steel and gain insights into the inhibition mechanism and kinetics, potentiodynamic polarization measurements were conducted for a blank solution and solutions containing various concentrations of *OML*, as shown in Figure 1. The curves consist of two regions: the cathodic region (more negative to −0.5 V vs. SCE), where the cathodic Hydrogen Evolution Reaction (HER) occurs, characterized by a moderate increase in the current density as the overpotential increases, and the anodic region (less negative to −0.5 V vs. SCE), where the anodic reaction (metal dissolution) takes place, resulting in a steeper current increase with potential [32].

The curves show that both the anodic and cathodic processes were inhibited when the OML inhibitor was added to the corrosive HCl environment; the whole polarization curves shifted to more negative potentials in the presence of *OML* in the solution compared to the blank solution. However, it can clearly be seen (Figure 1) that the cathodic currents significantly changed with increases in the *OML* concentration; the HER potential at, e.g., 0.001 mA cm^−2^, markedly shifts to higher overpotentials (i.e., more negative potentials) with increasing inhibitor concentration. This suggests that the *OML* inhibitor majorly hindered the cathodic HER reaction via adsorption at the metal surface, thus increasing the energy barrier for HER to occur and consequently hampering corrosion, mainly via the cathodic protection mechanism [33]. Notably, the polarization curves in the cathodic branch also indicate that HER is not controlled by diffusion.

Additionally, to quantify the underlying electrochemical processes, several important parameters were extracted by the analysis of the polarization curves, including the corrosion potential (*E_corr_*), the current density *(i_corr_*) corresponding to *E_corr_*, and the Tafel slopes of the anodic and cathodic reactions (*β_a_* and *β_c_*), as summarized in Table 2.

As shown in Table 2, *i_corr_*, which measures the corrosion rate on the metal surface, substantially decreases (from 620 to 453 µA cm^−2^) after the addition of *OML* in the solution. Moreover, the *i_corr_* value decreases monotonically with increasing *OML* concentration and reaches a minimum value of only 110 µA cm^−2^ at 500 ppm. Thus, the corrosion inhibition increases with increasing inhibitor concentration. This indicates that when the inhibitor molecules adsorb on the surface, they decrease the surface area available for corrosion reactions. Consequently, *i_corr_* decreases. This trend agrees with the weight loss method results. To explain this trend, several phenomena intervene, especially the adsorption mechanism and the reduction in surface active sites. Generally, there is a relationship between the adsorption of an inhibitor on the metal surface, blocking active sites, and the inhibitor concentrations following the Langmuir isotherm, with these sites being further blocked and inhibited as the concentration of adsorbed inhibitor on the metal surface increases, resulting in a decrease in *i_corr_* [14,33].

Further, the decrease in *i_corr_* is often expressed as the inhibition efficiency (*η_PDP_ %*), which quantifies the effectiveness of the inhibitor in reducing the corrosion rate compared to the blank sample and is calculated using Formula (3). It is evident from the data that an increase in *OML* concentration leads to a decrease in *i_corr_* values and, consequently, an increase in *η_PDP_* %. Specifically, at the concentration of 500 ppm, we found that *η_PDP_* % reached its maximum value of 82%.

To better understand the impact of *OML* on corrosion potential, the *E_corr_* shift in the presence of *OML* is a critical parameter that indicates its mechanism of action. According to the literature [34,35], when the *E_corr_* shift is less than 85 mV from the blank solution, this suggests that the inhibitor acts as a mixed-type inhibitor. In our case, as shown in Table 2, the presence of OML caused a slight shift in *E_corr_* (max. of 64 mV) towards more negative values, but it is less than 85 mV with respect to the blank. Based on this criterion, *OML* can be classified as a mixed-type inhibitor. Furthermore, a mixed-type inhibitor is supposed to inhibit both cathodic and anodic reactions to a certain extent; however, in our study, *OML* did not significantly inhibit the anodic reaction. Thus, we conclude that the inhibitor acts predominantly as a cathodic inhibitor. To unravel this effect, Tafel slope data provide insights into the mechanisms of the corrosion processes.

The values of *β_a_* and *β_c_* are affected by the inhibitor concentration; this means that the inhibitor affects the kinetics of both the anodic and cathodic reactions, resulting in changes in the slopes. Both the cathodic and anodic Tafel slopes changed after the addition of the OML inhibitor. For lower concentrations of the inhibitor, the change in the anodic Tafel slope *β_a_* is more pronounced than that of the cathodic Tafel slope *β_c_*; however, at the concentration of interest (500 ppm), both Tafel slopes change to a similar extent compared to the uninhibited solution. Therefore, the inhibitor functions as a mixed-type inhibitor. This indicates that the mechanism of the cathodic processes is slightly affected by the OML concentration and that this inhibitor functions by majorly hampering the cathodic HER reaction.

### 2.3. Electrochemical Impedance Spectroscopy (EIS)

**Figure 2:**
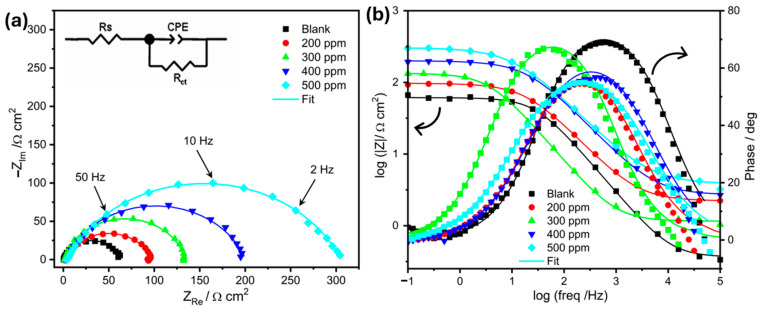
Nyquist plots (**a**) and Bode plots (**b**) of mild steel in 1 M HCl using various *OML* concentrations at 303 K. Inset of (**a**) is the equivalent electrical circuit used to fit EIS data.

To gain information about the formation of the protective film and the effectiveness of corrosion inhibitors, EIS is used to study the metal–solution interface by measuring the impedance (Z) over a range of frequencies [15,36]. Herein, the impedance spectra obtained from the uninhibited solution are compared to those in the solutions containing *OML*. For this, EIS experiments were conducted at OCP at a controlled temperature of 303 K after holding the potential at OCP for 30 min (Figure 2).

In all measurements, the Nyquist plots exhibit a depressed semicircular arc. The depressed capacitive loop may result from electrode surface inhomogeneity or surface roughness [14,37]. The EIS results feature a single time constant RC with charge transfer resistance (*R_ct_*) and double-layer capacitance (*C_dl_*), which means that steel corrosion is generally controlled by charge transfer at the steel–solution interface. Furthermore, the diameter of the semicircle increases with increasing inhibitor concentration, indicating that the impedance of the charge transfer at the inhibited substrate increases with increasing inhibitor concentration. This showcases that the addition of *OML* enhances the formation of a protective film that hinders charge transfer and thus promotes corrosion resistance. In addition, Figure 2b provides Bode plots to analyze the corrosion processes in depth, from which an increase in charge transfer resistance and a decrease in double-layer capacitance in the presence of *OML* can be observed.

To analyze the impedance spectra and determine such important interfacial parameters, the equivalent circuit shown in the inset of Figure 2a is used, where *R_s_* represents the solution resistance; *R_ct_* denotes the charge transfer resistance; and the constant phase element (CPE) is used to calculate *C_dl_*, which represents the interfacial capacitance. The resulting CPE impedance can be determined using the following Equation (1) [38]:

(1) $$Z_{CPE}=Q^{-1}\times {\left(j\omega \right)}^{-n}$$

where *Q* is the CPE constant, *ω* is the angular frequency with *ω* = 2*πf*, *f* is the frequency in Hz, *j*^2^ = −1 presents the imaginary number, and *n* is a CPE exponent. The *C_dl_* can then be calculated from Equation (2) [38]:

(2) $$C_{dl}=(Q^{1/n}{\left(R_{ct}\right)}^{1-n/n}$$

The impedance parameters obtained from the EIS curve fitting are summarized in Table 3. The inhibitory efficacy of each *OML* concentration is determined using Formula (4). In the blank solution, the Nyquist curves generally show lower *R_ct_* values due to higher corrosion rates, as displayed in Table 3. On the other hand, when *OML* is added as an inhibitor, an increase in *R_ct_* was observed, indicating reduced metal oxidation, and when different concentrations of OML were tested, a variation in *C_dl_* values could also be observed, including a change from *R_ct_* = 61.0 Ω cm^2^ and *C_dl_* = 86.6 µF cm^−2^ in the blank solution to *R_ct_* = 301.5 Ω cm^2^ and *C_dl_* = 47.0 µF cm^−2^ at 500 ppm. This implies that high concentrations lead to a significant increase in *R_ct_*, suggesting that there is an adsorption of *OML* molecules on the metal surface, forming a protective layer that hinders ion transport, leading to maximum inhibition efficiency [15,16]. The EIS results are consistent with the PDP and weight loss results.

For comparison with other plant extracts, an essential oil of *Eucalyptus botryoides* exhibited an inhibition efficacy of 76% at 600 ppm, which is less than our *OML* extract, which reached 80% at 500 ppm [39]. In another work, a commercial oil of eucalyptus was examined for the corrosion protection of mild steel in 1.0 M HCl solution, and an inhibitory efficiency of 84% was obtained at 500 ppm with the inhibitor [40]. Nevertheless, from this comparison, *OML* can be considered a high-performing inhibitor compared to the relevant inhibitors reported.

### 2.4. Effect of Temperature: Thermodynamics Evaluation

**Figure 3:**
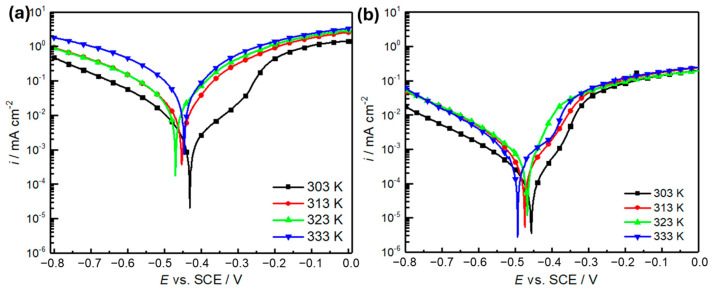
Polarization curves of mild steel in 1 M HCl at different temperatures in the absence (**a**) and presence of 500 ppm *OML* (**b**) inhibitor.

**Figure 4:**
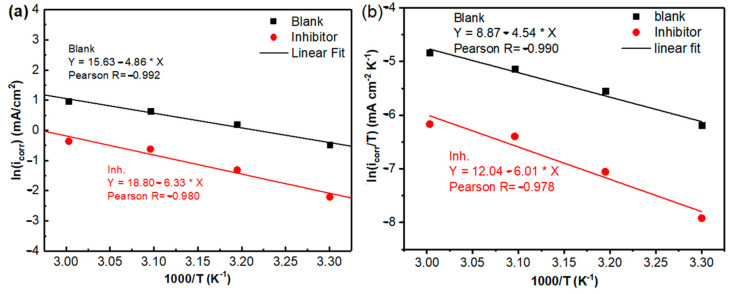
The different Arrhenius plots for mild steel in 1 M HCl (**a**) and the presence of 500 ppm *OML* (**b**).

The corrosion behavior of the metal under variable temperature was evaluated in the temperature range from 303 to 333 K using the PDP method (Figure 3). These tests are aimed at evaluating the absorption properties and activation variables associated with *OML* and their effect on the metal oxidation process. As demonstrated in Figure 4a, the increase in temperature clearly negatively affected the inhibition performance. Furthermore, to understand the effect of temperature on the attraction of *OML* to the metal surface, Figure 4b reveals the variations in the polarization curves with increasing temperature. It can clearly be seen that the corrosion current densities increase for the inhibited and uninhibited solutions with raising temperature. This effect can be interpreted by the partial desorption of inhibitor molecules from the surface and the increased mass transport of the corrosive species to the metal surface at higher temperatures. In other words, the adsorption equilibrium is shifted towards the desorption of the inhibitor, resulting in lower surface coverage and less inhibition. Additionally, the decrease in the inhibition efficiency with the increase in temperature can be related to the prominent temperature dependence of chemisorption. Generally, chemisorption is more affected by temperature in the context of corrosion compared to physisorption because the formation of chemical bonds requires overcoming an activation energy barrier, which is facilitated by higher temperature. Thus, elevated temperatures promote the chemisorption of oxygen or other reactive species, thereby increasing the rate of oxidation and corrosion.

For further quantification of this effect, Table 4 outlines the effect of temperature on the electrochemical parameters in the absence and presence of the *OML* (500 ppm) inhibitor in the solution. As shown in Table 4, the corrosion current intensity markedly increases from 620 μA cm^−2^ at 303 K to 2648 μA cm^−2^ at 333 K in the absence of *OML*. In the inhibited solution, *i_corr_* also increased at elevated temperatures; however, it did not exceed 700 μA cm^−2^ at 333 K. This indicates that despite the negative effect of high temperature on inhibitor performance, the *OML* molecules inhibited the acceleration of metal deterioration under such increased temperatures compared to the uninhibited solution. This is reflected in the inhibition efficiency, which does not drop below 70%. In general, the correlation between temperature and inhibition efficiency in an acidic environment is complex because various factors can affect the system, including adsorption and desorption processes, metal oxidation, and the stability of *OML* compounds.

To better understand the temperature effect on adsorption, the thermodynamics of the process were studied, starting from the Arrhenius equation (Figure 4a), to obtain the activation energy (*E_a_*) using the corrosion rate (*i_corr_*) of the mild steel, as follows:

(3) $$i_{corr}=Aexp\left(\frac{-E_{a}}{RT}\right)$$

where *A* is the Arrhenius constant, *R* is the ideal gas constant, and *T* is the absolute temperature.

To deduce other parameters, such as the activation enthalpy Δ*H_a_* and activation entropy Δ*S_a_*, a plot of *ln*(*i_corr_/T*) versus 1000*/T* was plotted in Figure 4b. Using the following relationship, the Δ*H_a_* value was calculated from the slope (−Δ*H_a_*/*R*), and the entropy Δ*S_a_* was calculated from the intercept:

(4) icorr=RTNhexp(ΔSaR) exp(−ΔHaRT)

where *N* is the Avogadro’s number, *R* symbolizes the universal gas constant, and *h* denotes Planck’s constant. In the context of understanding the impact of *OML* on corrosion inhibition, especially in the presence of *OML*, the corrosion process can be affected by the modification of the barrier energy. This behavior is evaluated by the *E_a_* value, which describes the minimum energy required for a chemical kinetic system to occur. As presented in Table 5, *E_a_* shows a higher value in the case of *OML* (53 kJ mol^−1^), suggesting that higher energy is required to initiate the corrosion process, thus demonstrating the effectiveness of the *OML* inhibitor. In agreement with previous reports [41,42], a protective layer of *OML* is formed on the metal surface that serves to modify the electrochemical properties of the system through a physical adsorption process, and the molecules adhere to the substrate through electrostatic bonds (physisorption).

The activation enthalpy value can be helpful to distinguish whether a system is endothermic or exothermic. The positive value of Δ*H_a_* reflects that the activation process is endothermic, i.e., absorbing heat from its surroundings. Furthermore, the obtained activation enthalpy value of 50 kJ mol^−1^ for *OML* indicates that the heat absorbed by the activation process is related to physical adsorption processes. This provides insight into the interaction between the mild steel surface and the *OML* molecules. Furthermore, the activation entropy Δ*S_a_* in the presence of *OML* was higher than that in its absence, suggesting that the activation process tends to have a high degree of disorder. This observation is consistent with many studies in the field of corrosion inhibition, in which inhibitors could disrupt any existing order caused by their aggregation [43], especially since *OML* is composed of various molecules, which can lead to an increase in the degree of disorder.

### 2.5. Adsorption Isotherm

**Figure 5:**
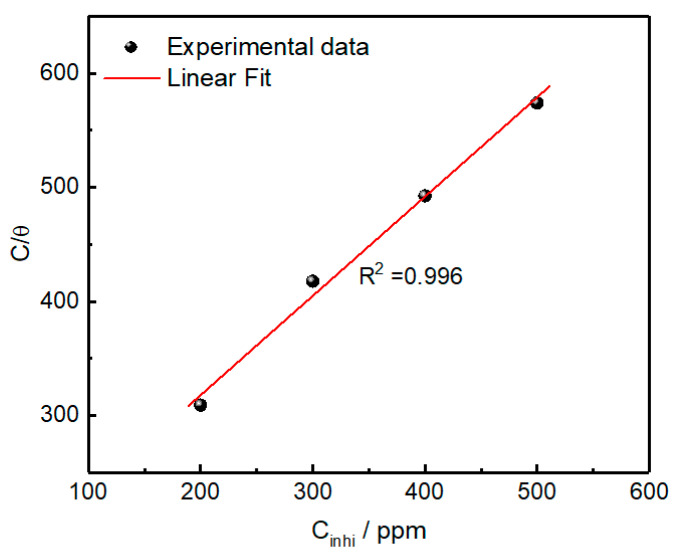
Langmuir adsorption isotherm of *OML* inhibitor on mild steel at 303 K.

Since adsorption is the main process in the inhibition action, the inhibitor molecules typically adsorb on the metal surface, displacing the already adsorbed water molecules. This adsorption is essential to shield the metal surface from the attack of corrosive species, thus hindering corrosion. The Langmuir adsorption isotherm (Equation (5)) was found to be the best fit of our experimental data, as shown in Figure 5.

(5) $$\frac{C_{inh}}{\;\theta }=\frac{1}{K_{ads}}+C_{inh}$$

where *θ* is the percentage surface coverage, *K_ads_* denotes the equilibrium constant of adsorption, and *C_inh_* is the inhibitor concentration. The Langmuir model revealed the best fit with a linear regression coefficient *R*^2^ of 0.996, demonstrating that the adsorption process obeys this isotherm. The intercept of the Langmuir plot was used to determine *K_ads_* and a value of 0.0069 ppm^−1^ was obtained, as presented in Table 6. The positive value of *K_ads_* along with a slope (0.87) close to unity suggest the monolayer adsorption of *OML* molecules on the electrode surface [44]. Although the standard free energy of adsorption (Δ*G_a_*), which can be determined from the *K_ads_* value, is commonly used to distinguish between physisorption and chemisorption, the corrosion expert Anton Kokalj has concluded that the standard free energy of adsorption may not be a reliable parameter to distinguish between chemisorption and physisorption, but rather the molecule–surface distance and electronic structure analysis, which can be simulated [45]. His report also revealed that physisorption is weak for small molecules, whereas for large molecules it may get stronger than chemisorption. This could be valid in our natural inhibitor, where the extract contains a mixture of large molecules, suggesting predominant physisorption. Moreover, the determination of Δ*G_a_* is challenging in our case due to the complex composition of the *OML* essential oil and the unknown average molecular weight of its constituents.

### 2.6. SEM-EDX Surface Imaging and Inhibition Mechanism

**Figure 6:**
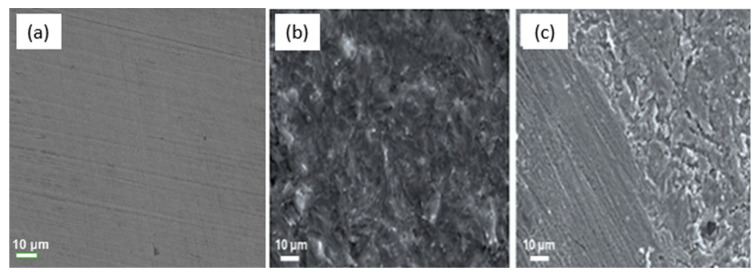
SEM micrograph of the mild steel surface examined prior to (**a**) and after 24 h of immersion in (**b**) 1 M HCl solution and (**c**) solution containing 500 ppm *OML*.

SEM and EDX techniques were utilized to investigate the contribution of *OML* molecules to metal corrosion prevention and possible surface damage. The SEM micrograph of the mild steel surface was examined after 24 h of immersion in 1 M HCl solution and in 1 M HCl solution containing 500 ppm *OML* (Figure 6). Figure 6a displays an SEM image of the polished mild steel surface that shows a flat surface without flaws, other than the typical polishing scratches caused by the sandpaper. Figure 6b shows the surface that was exposed to only 1 M HCl and reveals that the metal surface was affected by the acid solution with a homogeneous degradation pattern. Typically, this degradation occurs as a mixture of porous iron oxides, which lead to extensive corrosion of the underlying metal. In contrast, when the acid solution was modified with *OML* extract, the steel surface reveals less damage and maintains a fairly smooth appearance compared to the surface exposed to 1 M HCl solution (Figure 6c). These inspections suggest that *OML* participates in the formation of a protective layer on the metal surface, improving the resistance of the metal to the effects of corrosion.

**Figure 7:**
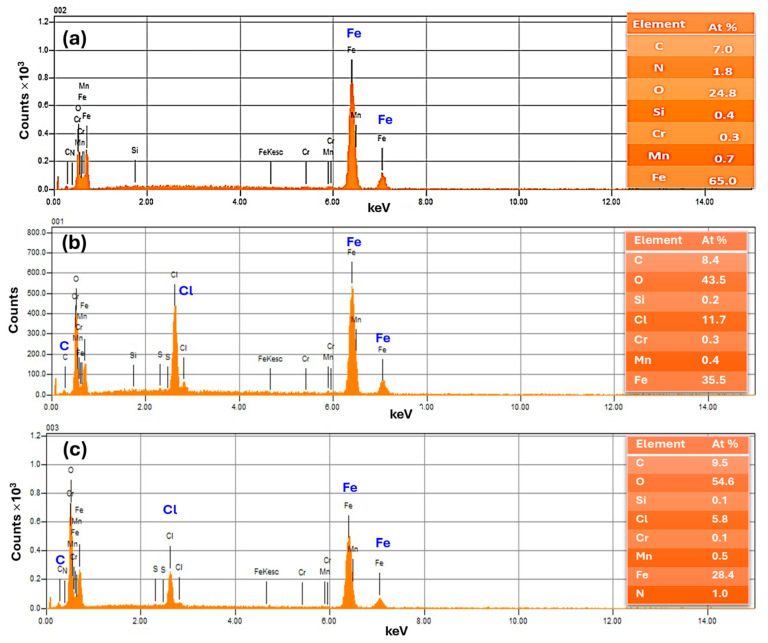
EDX spectra of mild steel surface examined prior to (**a**) and after 24 h of immersion (**b**) in 1 M HCl solution and (**c**) HCl containing 500 ppm *OML*. Inset shows the elemental analysis data.

**Figure 8:**
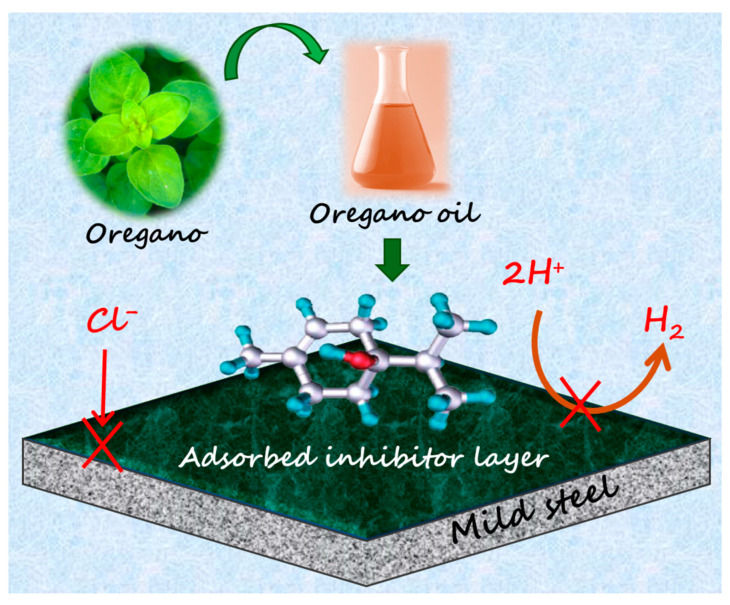
Schematic showing the adsorption of *OML* extract components on steel surface and protection against corrosion.

Furthermore, to determine the elemental composition of the samples, the EDX analysis technique was used; in addition, qualitative data on the elements present on the corroded surface are provided in Figure 7. The EDX spectrum of the naked mild steel surface (Figure 6a) reveals the appearance of mainly Fe and little signals of Mn and C, which agrees with the nominal composition of the coupon. No Cl signal was observed. By analyzing the EDX spectra of the immersed samples, the elements Fe, C, and Cl were identified, which can help to provide information on the nature of the species at the surface. In particular, when the solution was treated with the *OML* extract, the content in at% of Cl was decreased to 5.8% compared to 11.7% for the uninhibited sample, and Fe% was also decreased, as shown in the tables in the inset of Figure 7. On the other hand, the content of C was slightly increased, along with the emergence of a N signal. These results suggest the formation of an inhibitor layer of organic compounds that shields the steel surface from the attack of Cl. This result agrees with the above data from the various independent methods that suggest that the inhibition mechanism is based on the adsorption of *OML* extract molecules on the metal surface, thus forming a protective layer against the attack of corrosive species such as Cl and H^+^, as illustrated in Figure 8. This layer is mostly composed of oxygenated monoterpene compounds, which are characterized by the presence of π bonds and oxygen heteroatoms that can interact with the P-orbitals of Fe at the steel surface. In our case, the inhibitor layer minimizes the metal dissolution by retarding both the cathodic HER and the anodic metal dissolution; however, there is a predominant effect on the cathodic process. Furthermore, our organic inhibitor is rich in oxygenated functional groups that can form complexes or chelate with the metallic ions, such as Fe^2+^ and Fe^3+^, from the metal surface. These complexes can then adsorb on the surface and occupy the active sites, thus preventing corrosion.

## 3. Materials and Methods

### 3.1. Plant Collection

*OML* plants (leaves and stems) were collected in March 2021 Salé, Morocco. The plants were identified at the Department of Botany and Plant Ecology, University of Rabat, registered under the number RAB111871.

### 3.2. Extraction of Essential Oil

**Figure 9:**
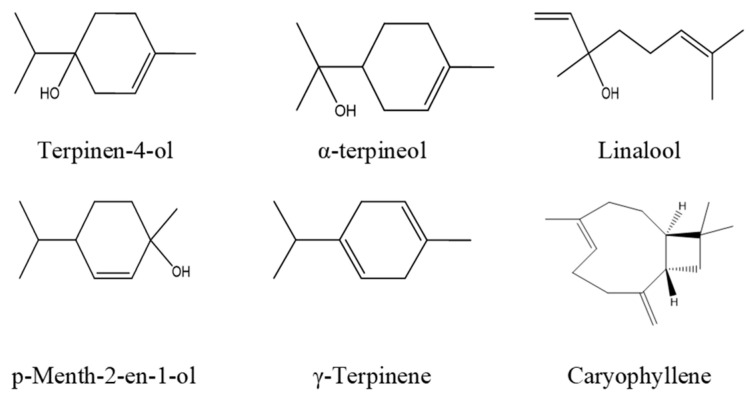
Chemical structure of the major components of *OML* oil extract.

Using a Clevenger-type apparatus, a hydrodistillation method was used to extract the essential oil. Briefly, in a flask half-filled with water, 150 g of the dried plant was added and the whole flask was brought to boil for four hours. The system included a condenser connected to a cooler, which cooled down the water vapor and essential oil, and an oil trap (Clevenger tube), where the essential oils separated from the water, after which the oil was transferred to a beaker or flask using a pipette. To preserve the oil, the essential oil was stored in dark bottles at 4 °C after being dried with anhydrous magnesium sulfate. According to the relevant literature on *OML* oil extracts, the major constituents of the OML oil extract are shown in Figure 9. We note that the concentration of these compounds can differ depending on the exact extraction conditions.

### 3.3. Preparation of Mild Steel Specimen

The used mild steel obtained from the ‘ThyssenKrupp’ Company, Essen, Germany, had an elemental composition (wt%) of 98.38% Fe, 0.28% C, 0.04% P, 0.05% S, and 1.25% Mn. Coupons (1 cm × 1 cm) were prepared and polished with 200–2000-grit abrasive paper in an ascending order, washed with distilled water, rinsed with acetone (VWR Chemicals, Darmstadt, Germany), and finally dried at room temperature.

### 3.4. Preparation of Solutions

The corrosive solution (1 M HCl) was prepared by diluting concentrated hydrochloric acid (37%) (VWR Chemicals, Germany) with distilled water. Various concentrations (200, 300, 400, and 500 ppm) of *OML* extract were dissolved in 1 M HCl at room temperature.

### 3.5. Weight Loss Method

The weight loss (WL) measurements were carried out by immersing the mild steel coupon (length × width × thickness of 1 cm × 1 cm × 0.2 cm) in 100 mL of 1 M HCL solution after weighing it. The weight loss was determined after immersing the mild steel coupons for 6 h in the presence and absence of the corrosion inhibitor at 303 K. The coupons were removed from the solution, washed with distilled water, dried, and re-weighed to calculate the WL. Each experiment was performed in triplicate. The results of the WL measurements were used to determine the *CR* (mg cm^−2^ h^−1^) and inhibition efficiency ($\eta_{WL}\%$) by applying Equations (6) and (7), respectively [16].

(6) $$C_{R}=\frac{\Delta m}{S\times t}$$

(7) $$\eta_{WL}\%=(\frac{C_{R}^{{}^{\circ}}-C_{R}^{inh}}{C_{R}^{{}^{\circ}}})\times 100$$

where $C_{R}^{{}^{\circ}}$ and $C_{R}^{inh}$ represent the corrosion rate in the absence and presence of the *OML* inhibitor, respectively, *t* is the immersion time (h), and *S* is the surface area of the mild steel coupon (cm^2^).

### 3.6. Electrochemical Monitoring

Electrochemical measurements were performed using a Voltalab PGZ 100 potentiostat (Radiometer analytical, Alsace, France) controlled by “Volta Master 4” software (Version 7.8). The electrochemical setup consists of a three-electrode arrangement with a mild steel sheet with an exposed area of 1 cm^2^ as the working electrode, a saturated calomel electrode (SCE, Metrohm, Barendrecht, The Netherlands) as the reference, and a platinum (Pt, Ögusse, Vienna, Austria) electrode as the counter electrode. All electrochemical experiments were performed after 30 min of recording open circuit potential (OCP). All measurements were conducted in triplicate. EC-Lab software (V10.34) was then used to retrieve the electrochemical parameters. Furthermore, the potentiodynamic polarization (PDP) tests were used to evaluate the corrosion phenomenon and the efficiency *η_PDP_* % of *OML* (Equation (8)). The anodic and cathodic polarization curves were acquired from −800 mV to 0 mV vs. SCE at a constant scan rate of 0.5 mV s^−1^.

(8) $$\eta_{PDP}\%=\frac{i_{corr}-i_{corr}^{o}}{i_{corr}}\times 100$$

where $i_{corr}$ and $i_{corr}^{o}$ are the corrosion current densities of the metal surfaces in 1 M HCl with and without *OML*, respectively.

EIS measurements were performed at OCP using alternating amplitude (peak-to-peak) signals of 10 mV in a frequency range of 10 kHz to 100 mHz. The efficiency of *OML* was evaluated using Equation (9).

(9) $$\eta_{EIS}\left(\%\right)=\frac{R_{ct}^{i}-R_{ct}^{0}}{R_{ct}^{i}}\times 100$$

where $R_{ct}^{i}\;\mathrm{and}\;R_{ct}^{0}$ are the charge transfer resistance of mild steel in the presence and absence of *OML*, respectively.

### 3.7. Surface Morphology Analysis

The morphology and composition of the samples were examined by scanning electron microscopy (SEM) using a JEOL-JSM-IT100 equipped with a JEOL-made EDX detector, Tokyo, Japan. The morphology and elemental composition of the mild steel were characterized after 24 h immersion in 1 M HCl without and with the addition of the optimum concentration of *OML* (500 ppm).

## 4. Conclusions

The essential oil of *Origanum majorana* L. (*OML*) was extracted and applied as a bio-based corrosion inhibitor for mild steel in 1 M HCl medium. The results underlined the potential anticorrosive effect of *OML* using weight loss, potentiodynamic polarization (PDP), and EIS methods. The *OML* extract matrix was found to significantly prevent and slow down the damage to mild steel caused by the HCl solution. For instance, the weight loss data showed a reduced corrosion rate, reaching a minimum of 0.11 mg cm^−2^ h^−1^ at a concentration of 500 ppm *OML*. In addition, the PDP method confirmed the inhibition effect, where the corrosion current density decreased significantly from 620 to 110 µA cm^−2^ and both the anodic and cathodic processes were affected, so that the inhibitor functioned as a mixed-type inhibitor with cathodic dominance. The EIS results revealed that the charge transfer mechanism is the main mechanism controlling the corrosion process, and this charge transfer was reduced in the presence of *OML*. Furthermore, the temperature effect was also evaluated for the *OML* inhibitor, and the thermodynamic values of the activation process were deduced, which allowed us to conclude that the adsorption process is related to physical adsorption and the adsorption process follows the Langmuir isotherm. Furthermore, SEM and EDX analyses indicated that *OML* constituents can interact with the metal surface, forming a protective layer that prevents corrosion. Insights into the mechanism of action of this extract-based inhibitor were provided. Nonetheless, a main limitation of this research work is the difficulty to identify which compound in the extract is responsible for the corrosion-inhibition effect. Separating the bioactive compounds of the extract and testing them individually could be a possible approach, if the testing cost of all of these components is still reasonable. In addition, further research is needed to determine whether such individual compounds are capable of inhibiting corrosion on their own or in combination with others (synergy). In summary, the expanding field of plant extracts not only addresses environmental issues, but also brings corrosion research forward by providing improved protection through efficient natural inhibitors.

## Figures and Tables

**Table 1:** Corrosion rate and inhibition efficiency results of weight loss measurements.

*C_inh_* (ppm)	*CR* (mg cm^−2^ h^−1^)	*η_WL_* %
0	0.85 ± 0.08	--
200	0.30 ± 0.04	64.7
300	0.24 ± 0.04	71.8
400	0.16 ± 0.03	81.2
500	0.11 ± 0.01	87.1

**Table 2:** Electrochemical parameters of mild steel in 1 M HCl containing various concentrations of *OML* at 303 K.

*C_inh_* (ppm)	*E_corr_* (mV vs. SCE)	*i_corr_* (µA cm^−2^)	*β_c_* (mV dec^−1^)	*β_a_* (mV dec^−1^)	*η_PDP_* (%)
0	−420 ± 2	620 ± 3	−190 ± 2	140 ± 3	--
200	−465 ± 2	453 ± 2	−197 ± 5	101 ± 4	27
300	−460 ± 3	288 ± 4	−173 ± 4	88 ± 3	54
400	−472 ± 2	230 ± 4	−165 ± 3	91 ± 3	63
500	−484 ± 3	110 ± 4	−156 ± 5	109 ± 4	82

**Table 3:** EIS parameters of mild steel in 1 M HCl using various concentrations of OML at 303 K.

*C* (ppm)	*R_s_* (Ω cm^2^)	*R_ct_* (Ω cm^2^)	*Q* (μs^n^ Ω^−1^ cm^−2^)	*n*	*C_dl_* (µF cm^−2^)	*χ* ^2^	*η_EIS_* (%)
0	0.4 ± 0.5	61 ± 4	170 ± 4	0.87 ± 0.1	87 ± 4	0.0046	-
200	2.2 ± 0.5	95 ± 3	226 ± 3	0.79 ± 0.1	83 ± 3	0.0036	36
300	1.2 ± 0.5	115 ± 3	221 ± 3	0.77 ± 0.1	74 ± 3	0.0066	47
400	2.7 ± 0.8	199 ± 4	109 ± 4	0.79 ± 0.1	38 ± 4	0.0046	69
500	3.8 ± 1	302 ± 2	141 ± 2	0.74 ± 0.1	47 ± 2	0.0009	80

**Table 4:** Effect of temperature on the electrochemical parameters of mild steel in 1 M HCl and the presence of 500 ppm *OML*.

*T* (K)	*i_corr_* (μA cm^−2^)		*θ = η*/100	*η_PDP_* (%)
	Blank	*OML*		
303	620 ± 5	110 ± 4	0. 82	82
313	1215 ± 9	270 ± 5	0.78	78
323	1891 ± 10	539 ± 4	0.71	71
333	2648 ± 10	698 ± 3	0.74	74

**Table 5:** Thermodynamic parameters for mild steel in 1 M HCl and the presence of 500 ppm *OML*.

*C_inh_* (ppm)	*E_a_* (kJ mol^−1^)	Δ*H_a_* (kJ mol^−1^)	Δ*S_a_* (J mol^−1^K^−1^)
0	40 ± 2	38 ± 2	−124 ± 2
500	53 ± 3	50 ± 3	−97 ± 3

**Table 6:** Langmuir adsorption isotherm parameters for the adsorption of *OML* inhibitor.

	Slope	Intercept (ppm)	*K_ads_* (ppm^−1^)	*R* ^2^
*OML* inhibitor	0.87	144.0	0.0069	0.996

## Data Availability

Data are contained within the article.
